# Study on the Remediation of Cd Pollution by the Biomineralization of Urease-Producing Bacteria

**DOI:** 10.3390/ijerph16020268

**Published:** 2019-01-18

**Authors:** Xingqing Zhao, Min Wang, Hui Wang, Ding Tang, Jian Huang, Yu Sun

**Affiliations:** School of Environmental and Safety Engineering, Changzhou University, Changzhou 213164, China; mwangcz@163.com (M.W.); wh1016654221@163.com (H.W.); 18551352522@163.com (D.T.); huangjian08170@163.com (J.H.); sunyu666wa@163.com (Y.S.)

**Keywords:** biomineralization, urease, heavy metal, sequential extraction, bioremediation

## Abstract

Cadmium (Cd) is a highly toxic metal that can affect human health and environmental safety. The purpose of this study was to research the removal of Cd from an environmental perspective. In this article, four highly urease-active strains (CZW-2, CZW-5, CZW-9 and CZW-12) were isolated from an abandoned mine and their phylogenetic trees were analyzed. The maximum enzyme activities, the mineralized precipitate and the removal rates of these strains were compared. The results showed that CZW-2 had the highest urease activity at 51.6 U/mL, and the removal rates of CZW-2, CZW-5, CZW-9 and CZW-12 after 120 h were 80.10%, 72.64%, 76.70% and 73.40%, with an initial concentration of Cd of 2 mM in the Cd precipitation experiments. XRD (X-ray diffractometer), EDS (Energy dispersive spectrometer) and FTIR (Fourier transform infrared spectroscopy) analysis indicated that the mineralized precipitate was CdCO_3_. SEM (Scanning electron microscopy) analysis revealed that the diameter of the oval-shaped mineralized product ranked from 0.5 to 2 μm. These strains were used to remedy Cd-contaminated soil, and five different fractions of Cd were measured. Compared with the control, the results of spraying pre-cultured strains containing 2% urea to remove Cd from contaminated soils showed that the exchangeable fraction of Cd decreased by 53.30%, 27.78%, 42.54% and 53.80%, respectively, whereas the carbonate-bound fraction increased by 55.42%, 20.27%, 39.67% and 34.36%, respectively, after one month. These data show that these strains can effectively reduce the bioavailability and mobility of Cd in contaminated soils. The results indicate that biomineralization based on the decomposition of substrate urea can be applied to remedy heavy contaminated soil and water.

## 1. Introduction

Contamination of natural habitats by heavy metals through industrial and agricultural activities has resulted in serious excess of Cd content in the environment in China [1]. Cd is considered as a strong carcinogenic element and can cause damage to human health through the food chain [2]. Because of this, the hazards of Cd are related to its different oxidation states, mobility and bioavailability [3,4]. Therefore, remediation of Cd-contaminated water and soil is highly desired.

Considering the economic benefit and environmental protection, traditional repair methods are costly and only suitable for small areas, as they can greatly disturb the environment and are likely to cause secondary pollution [5]. Phytoremediation is an effective method to remove Cd contamination, but the plant’s growth conditions depend on many elements, such as moisture, salinity, temperature and climate [6]. However, the use of the principle of biomineralization to remediate heavy metals from contaminated environments is an in situ bioremediation technology that is suitable for various geographical locations and environmental conditions and has good application prospects [7,8]. It is an emerging direction of repair and has attracted attention due to its characteristics [9]. Therefore, more researchers are increasingly interested in using microorganisms to remedy heavy metal contamination. Zhao et al. [10] reported that microbe GZ-22 has the ability to produce urease and can effectively remove Cd under the condition of adding substrates.

Urease (urea amide hydrolase, EC3.5.1.5) commonly exists in many microorganisms [11], and urease-producing bacteria have the ability to promote the formation of carbonate minerals and are diverse and widely distributed. The basic principle of the biochemical pathway is that bacteria produce urease to hydrolyze urea into NH^4+^ and CO_3_^2−^, and NH^4+^ releases NH_3_, which increases the average pH until the NH^4+^/NH_3_ and HCO_3_^−^/CO_3_^2−^ ratios achieve equilibrium [12]. Under alkaline conditions, the metal ions combine with CO_3_^2−^ to form a precipitation that could convert the soluble heavy metals into insoluble carbonate mineral crystals. Microorganisms immobilize heavy metals in mineral crystal lattices or convert heavy metals into insoluble minerals through metabolism to mineralize heavy metal ions, thereby reducing their mobility and bioavailability. In short, urease-producing bacteria can promote mineral precipitation, which can play a role in removing and passivating metals in soil and waste [13,14]. Therefore, screening and preserving the native urease-producing mineralization strains maintains the original ecological environment of in situ remediation of soil and may result in the more uniform distribution of bacteria, making it more suitable for practical engineering applications.

At present, there are many studies focusing on the hydrolysis of urea by urease-producing bacteria to produce carbonate precipitates. The previous studies revealed that researchers observed the differentiation of the physiological and biochemical characteristics of urease-producing bacteria from different regions [8,15,16], and explained that urease-producing bacteria had a significant effect on removing heavy metals [11]. Recently, many urease-producing microorganisms have removed heavy metals under certain conditions, including bacterial species of *Bacillus*, *Sporosarcina*, *Enterobacter*, and *Pseudomonas* [17,18,19,20]. Annu et al. [20] reported that *Saccharomyces cerevisiae* has the capability to uptake 65–82% of Cd within 30 days when the pH of the soil was maintained at 5.5. Kang et al. [21] reported that KJ-46 has high removal rates (68.1%) of Pb under the initial concentration of 7.2 mg/L with excellent adaptation abilities and promising remediation efficiency. However, most studies explored the removal efficiency of heavy metals by a single microorganism [6,22], and showed that high urease activity is beneficial to increase the removal rate of heavy metals. Despite these studies, we still know little about the crystallinity, size, bioavailability and stability of Cd mineralization precipitates. 

In the present study, the aim was to evaluate the removal rates of Cd^2+^, the efficiency of microorganism reduced bioavailability of heavy metals, and the differences in the carbonate crystal structure. Meanwhile, microbial reduction of heavy metal mobility and bioavailability was explored from two aspects, such as immobilization of toxic metals in vitro and for soil remediation. In addition, the relationship between the urease-producing ability of the strain and the immobilization of toxic metals, crystallinity and stability of precipitates is discussed. In order to get the effect of biomineralization, the precipitates were characterized by XRD (X-ray diffraction), FTIR (Fourier transform infrared spectroscopy), SEM-EDS (Scanning electron microscopy and energy dispersive X-ray spectroscopy), and five different fractions of Cd were measured by the Tessier sequential extraction procedure. This study provides experimental results and a theoretical basis for further application of microorganisms to mitigate heavy metal pollution in contaminated soils and wastewater.

## 2. Materials and Methods

### 2.1. Screening Strains

Five heavy metal contaminated soil samples were randomly collected from the Shizishan mining area, Tongling, Anhui Province, China. The characteristics and metal concentrations of the soil samples were as follows: pH 5.60; 46.70 mg/kg Pb; 180.10 mg/kg Zn; 450.20 mg/kg Cu; and 5.27 mg/kg Cd. Urease-producing strains were isolated by serially diluting 1 g of soil with sterile water, and samples were shaken at 170 rpm for 6 h. Then, the dilute soil suspensions were transferred onto a urea agar base (5.0 g/L beef extract, 10.0 g/L peptone, 5.0 g/L NaCl, 60.0 g/L urea, 30 mL/L phenolphthalein), and cultured in an incubator at 30 °C for 48 h. Finally, the colonies that changed the color of the medium from yellow to pink were selected and further purified. The lowest concentration of the metal that totally inhibits bacterial growth was considered the minimum inhibitory concentration (MIC) [23]. The isolated urease-producing bacteria were rescreened by the streak plate method on Cd containing nutrient agar plates, and the Cd concentrations were continuously increased (25–400 mg/L). Plates were incubated and observed for bacterial growth at 30 °C for 48 h. 

### 2.2. Identification Based on Molecular Characterization

Genomic DNA was extracted from the logarithmic phase of bacterial cells by alkaline lysis method. The primers were designed and synthesized according to the most conserved sequence in bacterial 16S rDNA; forward primer 27F: 5’-AGAGTTTGATCCTGGCTCAG-3’, reverse primer 1492R: 5’-TACCTTGTTACGACTT-3’ (synthesized by Sangon Biotech Co., Ltd., Shanghai, China). The PCR amplification was performed using a thermocycler as described by Arias and Achal [14,24]. 16S rDNA amplification products were sent to Sangon Biotech (Shanghai) Co., Ltd. for sequencing. Finally, the resulting sequences were submitted to GenBank for registration, and BLAST (Basic Local Alignment Search Tool) analysis was performed in the GenBank database [6]. Using ClustalX 1.8 (NCBI, Bethesda, MD, USA) for comparison and the neighbor-joining analysis of the phylogenetic tree generation system by the MEGA 5.0 software (http://www.megasoftware.net) with the Kimura 2-parameter model, the phylogenetic tree was tested by the bootstrap method (1000 replicates) [25]. The 16S rDNA gene sequences of the strains were obtained and uploaded to GenBank with the following accession numbers: MG866078 (strain CZW-2), MG866079 (strain CZW-5), MG866080 (strain CZW-9) and MG866081 (strain CZW-12).

### 2.3. Enzyme Activities

During the experiment, the optical density of cultured bacteria was measured at 600 nm and used as an indicator of biomass density. In the absence of calcium ions, the urease activity was confirmed at regular time intervals by the electrical conductivity variation. The change of conductivity was measured with a conductivity meter according to the studies of Whiffin and Whiffin et al. [26,27]. Within the measured range of activities, our measurements of conductivity change correlated with a hydrolysis activity of 11.1 mM urea per minute. The bacterial urease activity was equal to the change in the conductivity of the bacteria urea mixture (ms/cm/min) during the measured time, multiplied by the dilution multiple, and multiplied by 11.1.

### 2.4. Cd Precipitation Experiments

The composition of LB (Luria-Bertani) liquid medium was 5.0 g/L beef extract, 10.0 g/L peptone, 5.0 g/L NaCl, pH 7.0. Taking 79 mL LB liquid medium into each beaker flask, all of the media were autoclaved at 121 °C for 20 min, 1 mL of the logarithmic phase bacteria was added in the liquid medium, the initial optical densities (OD_600_) were all kept at 0.03, and 10 mL of 20 mM CdCl_2_·2.5H_2_O solution and 20.0 g/L of urea solution filtered through 0.22 µm filter were added, respectively. Then, the samples were incubated (170 rpm) at 37 °C for 120 h. Samples were taken at regular time intervals and the pH was measured. The concentrations of Cd^2+^ in the samples were measured using an atomic absorption spectrometer (novAA300, Analytik Jena AG, Jena, Germany). 

### 2.5. SEM and EDS Analyses of the Precipitates

The precipitate collected by centrifugation (8000 rpm, 5 min) was first fixed with 2.5% glutaraldehyde fixation solution for 1.5 h. Then, the precipitate collected after centrifugation was formed by the addition of ethanol by 30%, 50%, 70%, 90% and 100% of the gradient to dehydrate for 15–20 min [28], and the resulting precipitate was dried for 6 h in a lyophilizer (FD-1A-50, Beijing Boyikang Laboratory Instrument Co., Ltd., Beijing, China). An SEM (JSM-6360 LA, Japan Electron Optics Laboratory Co., Ltd, Tokyo, Japan) was used to observe the structural morphology of the mineral precipitate at a stable voltage of 15 kV and EDS (JSM-6360 LA, Japan Electron Optics Laboratory Co., Ltd, Tokyo, Japan) performed a detailed elemental analysis of the mineral precipitation components.

### 2.6. XRD and FTIR Analyses

The mineral composition of the precipitate was tested by an X-ray diffractometer (XRD, D-MAX2500, Japan Corporation Co., Ltd., Tokyo, Japan). The samples were fully dried and then ground to a size of 300 mesh (48 μm) before they were measured on the instrument. The test conditions included a voltage of 40 kV, a tube current of 100 mA, CuKα = 1.54056 Å, and a scan angle (2*θ*) ranging from 5° to 80° in steps of 0.02°. FTIR was used to analyze the specific functional groups in the precipitated samples (Nicolet Avatar 370 (iS10), Thermo Nicolet Corporation, Madison, WI, USA). The infrared spectrum of the precipitate was determined by the KBr pellet pressing method, in which a 1-mg treated sample was finely mixed with 150 mg of dried KBr, and pressed at 10 t·cm^−2^ for 2 min. The FTIR spectrometer had a scanning wavenumber range from 500 to 4000 cm^−1^ and a scanning accuracy was 4 cm^−1^.

### 2.7. Cd-Contaminated Soil Remediation

Soils were taken from clean farmland at the National Rice Field Demonstration Base in Danyang City, China. The soil samples were exposed to heavy metal Cd, with an initial concentration of 5.10 mg/kg. Cd-contaminated soils were autoclaved at 121 °C and were taken in a square container (20 cm × 10 cm × 10 cm). The isolated bacteria were cultured in LB liquid media about 24 h before irrigating the Cd-contaminated soil. Soil was sprayed with the bacteria solution (100 mL) containing 2% urea evenly at an interval of 24 h for 2 weeks and was then left for 1 month at 30 °C. After 1 month, the mineralized soil samples were collected and used for follow-up experiments. Control experiments were similarly carried out without bacteria.

### 2.8. Sequential Extraction of Cd

The Cd in the Cd-contaminated soil was measured by the Tessier sequential extraction procedure for 5 different fractions: exchangeable, carbonate-bound, Fe-Mn oxides-bound, organic matter-bound, and residual forms [29]. The extracts of 5 different fractions at a constant volume were measured using an atomic absorption spectrometer (novAA300, Analytik Jena AG, Jena, Germany).

## 3. Results and Discussion

### 3.1. Screening and Identification of Bacteria

The urea agar plates were used to choose positive strains authenticated by pink color resulting from the hydrolysis of urea products. After repeated screening, four strains of Cd-resistant and urease-producing strains were finally isolated, and the MIC value of the bacteria was 350 mg/L; these strains were coded as CZW-2, CZW-5, CZW-9 and CZW-12.

**Figure 1:**
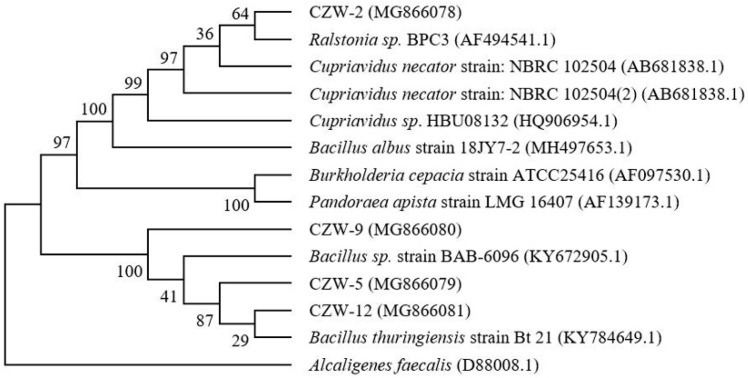
Neighbor-joining tree based on bacterial (CZW-2, CZW-5, CZW-9, CZW-12) 16S rDNA gene sequence data from different isolates obtained in the heavy metal-contaminated soil. Numerical values indicate bootstrap percentile from 1000 replicates. The 16S rDNA sequences of *Alcaligens faecalis, Burkholderia cepacia* and *Pandoraea apista* were included as outgroups.

The sequencing results of the strains were uploaded to the Biotechnology Information Bank and compared with the known sequences in GenBank [6]. The phylogenetic tree (Figure 1) reveals that strain CZW-2 corresponds to *Cupriavidus* sp. with 98% certainty; CZW-5, CZW-9 and CZW-12 correspond to *Bacillus* sp. with 100% certainty. These strains were with strong heavy metal resistance and the capacity for biodegradation, and widely used to remove heavy metals from contaminated environments [17,30]. The genus *Cupriavidus* is a *β*-proteobacterium that thrives in some of the harshest environments [31]. In previous studies, Mergeay et al. [32] had isolated *Cupriavidus metallidurans* CH34 from industrial sites polluted by industrial wastes rich in toxic heavy metals, and found that it has strong heavy metal tolerance. Zhu et al. [8] isolated *Bacillus cereus* NS4 from the industrial soil of a battery factory, and utilized it on a large scale to remedy nickel contaminated soil.

### 3.2. Enzyme Activities

**Figure 2:**
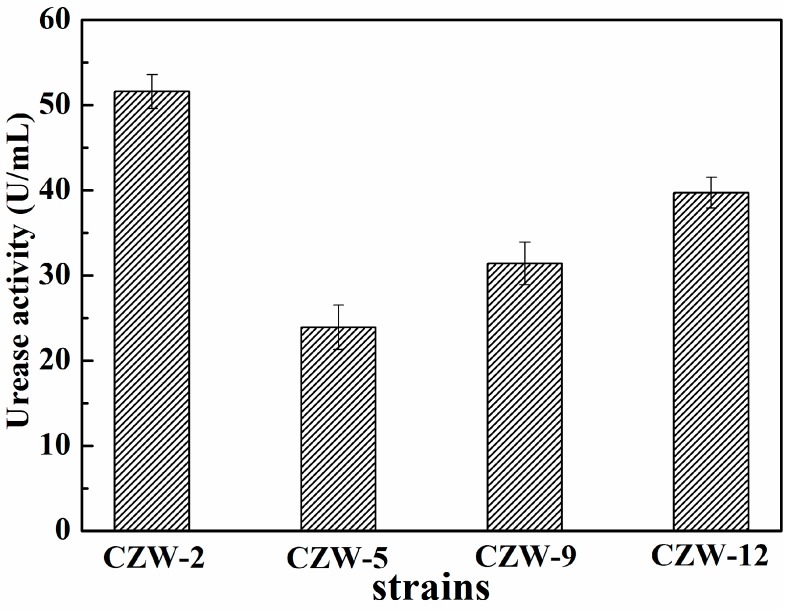
Urease activity of four isolated strains.

Urease is the key to the formation of precipitates of calcium carbonate in a medium containing the substrate urea and a calcium source [33]. The urease activity of four strains is shown in Figure 2. Among the isolated strains, CZW-2 showed the highest urease activity was 51.6 U/mL. The urease activities of CZW-5, CZW-9 and CZW-12 were 23.9 U/mL, 31.4 U/mL and 39.7 U/mL, respectively. The above results were analyzed by the one-way ANOVA method in SPSS 22.0 (IBM, New York, NY, USA), and there were significant differences among all the data (*p* < 0.05). During the bacterial growth period, the urease activities of CZW-2 were obviously an advantage compared to the other three strains. 

### 3.3. Cd Precipitation Experiments

**Figure 3:**
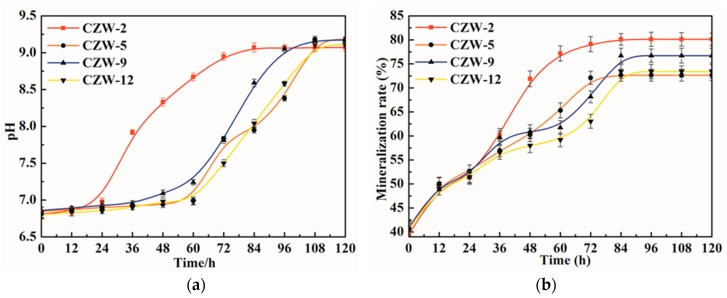
Analysis of the pH evolution (**a**) and removal rate (**b**) in the Cd precipitation experiments in Cd^2+^-LB broth from 0 to 120 h.

Previous studies have shown that strains have higher urease activity under neutral or alkaline conditions [34,35]. In this study, the strains were added to a medium containing urea and Cd^2+^ at conditions of pH = 7 and 37 °C for Cd^2+^ precipitation (Figure 3). Figure 3a shows the change in pH during the process of Cd precipitation for four strains. Compared with the other three bacteria, CZW-2 has a rapid increase in pH and a short delay time for the strain to enter the logarithmic growth stage. At this time, the bacteria multiply in the culture medium and produce urease to hydrolyze urea, thereby rapidly generating NH_4_^+^ and CO_3_^2−^ to cause the pH increase, accelerating the biochemical reaction in the liquid environment [36]. The other three strains of CZW-5, CZW-9 and CZW-12 exhibit similar urease activity, pH value and removal rate. During 0–48 h, the pH of the mineralization system exhibits a small increase. However, during 48–108 h, the pH of the mineralization system increases by approximately 1.6, reaching values of 9.15, 9.17 and 9.11, respectively. After 108 h, the four strains basically reach a stable phase at the same time. In short, strain CWZ-2 had the highest efficient hydrolysis of urea, and significantly better than the other three strains.

The removal rates of the four strains on the Cd precipitation experiments are different (Figure 3b). The initial total Cd concentration is approximately 2 mM. Within 120 h, strain CZW-2 has a Cd^2+^ removal rate of 80.10%. The other three strains of CZW-5, CZW-9 and CZW-12 exhibit close efficiency in the removal rate. The removal rates continuously increased within 0–84 h and reached the highest rates of 72.64%, 76.70% and 73.40% at 84 h, respectively. Compared with the other three strains, strain CWZ-2 had the highest removal rate. The pH of the system increased but the removal rate remained stable from 84 h to 96 h (Figure 3b),. The reason for this is perhaps that when the number of bacteria or functional groups and other binding sites on the bacterial cell wall have all been occupied by metal ions, the existing urease in solutions still hydrolyzes the urea [37]. There can be many similar mechanisms for urease-producing bacteria to mineralize heavy metals. Choi et al. [38] screened *Ralstonia* sp. and *Bacillus* sp. strains from soil contaminated with heavy metals and diesel oil to remediate the metal contamination of Cd, Cu and Pb with a removal efficiency of more than 90%. Bacteria belonging to *Bacillus* are able to hydrolyze urea into NH_4_^+^ and CO_3_^2−^ to precipitate calcite in their microenvironment [39,40]. Cheng et al. [41] determined that the free Cd^2+^ in soil can combine with the CO_3_^2−^ formed by the hydrolyzed urea by the strain to form crystals.

### 3.4. XRD Analysis

**Figure 4:**
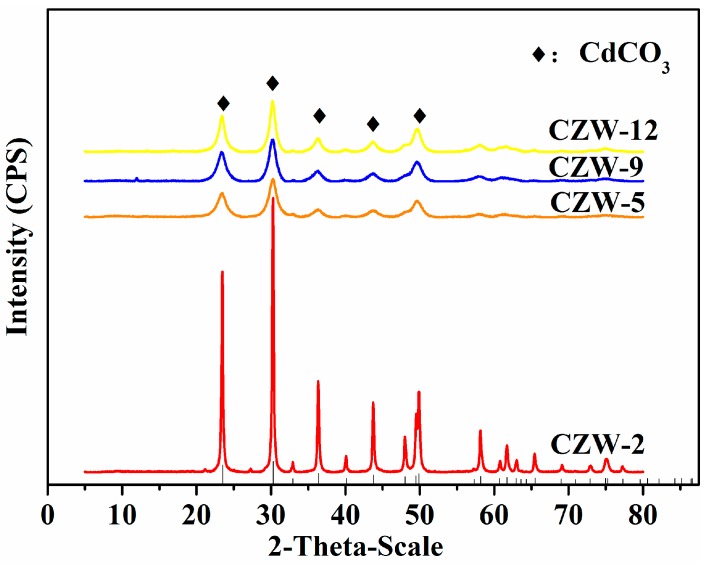
XRD analysis of the precipitates produced by CZW-2, CZW-5, CZW-9 and CZW-12. Identified peaks: CdCO_3_ (♦).

To qualitatively analyze the precipitated substances, XRD analysis was carried out. The XRD spectra of the precipitated substances produced by CZW-2, CZW-5, CZW-9 and CZW-12 mineralization were observed (Figure 4). The XRD spectra revealed that the mineralized products were CdCO_3_. The peak intensity decreased in the following order: CZW-2 > CZW-12 > CZW-9 > CZW-5. The main CdCO_3_ peak of CZW-2 was much higher than the other three strains, and showed that the degree of crystallinity of CZW-2 was better than that of the other three. This relationship indicates that the higher the peak, the smaller the peak width, and the better the crystallinity [42].

### 3.5. SEM and EDS Analyses

**Figure 5:**
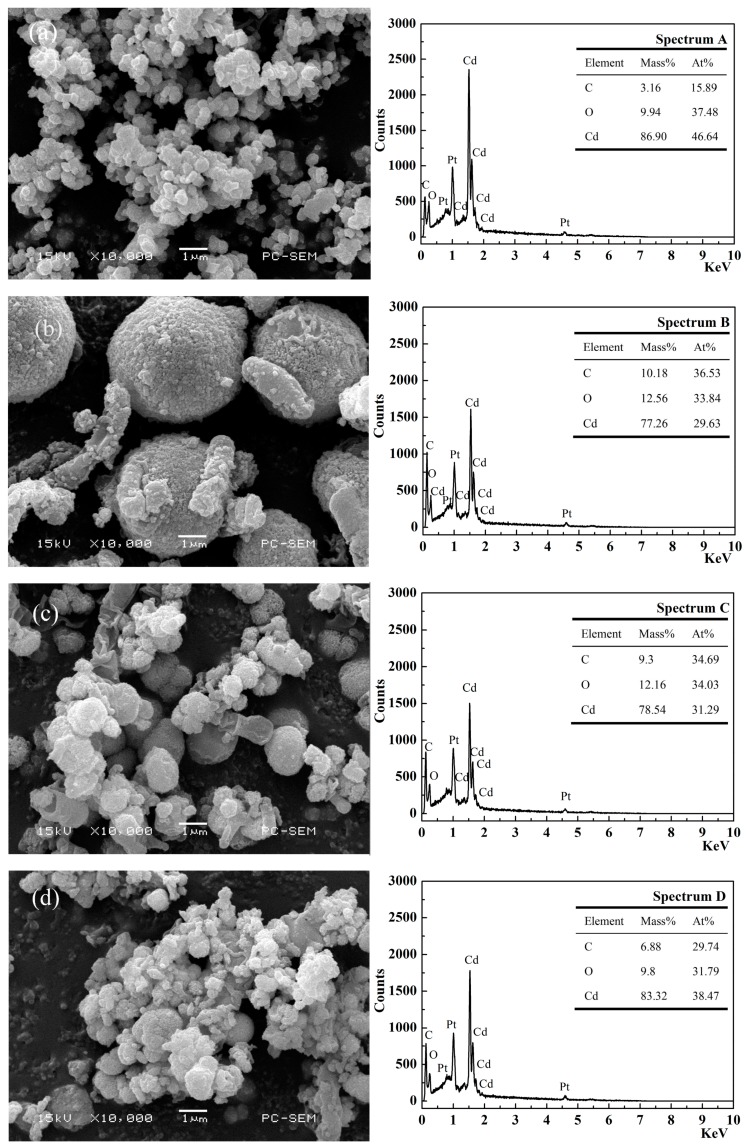
Scanning electron microscopy and energy dispersive X-ray spectroscopy (SEM-EDS) analysis of the precipitates produced by CZW-2, CZW-5, CZW-9 and CZW-12. (**a**): CZW-2, (**b**): CZW-5, (**c**): CZW-9, (**d**): CZW-12. Elemental composition displayed in the graphs indicates that they are composed of Cd, C and O.

Bacterial activity controls the mineralization process to control mineral nucleation growth [43,44], and the types of the precipitated mineral that are affected by the microbial species [45]. The precipitations mineralized by strains CZW-2, CZW-5, CZW-9 and CZW-12 were analyzed by scanning electron microscopy and energy dispersive X-ray spectroscopy (SEM-EDS) (Figure 5). The mineral precipitated by strain CZW-2 was an oval-shaped mineralized product, and its diameter was approximately 0.5 μm. The SEM images showed that the number of crystals was many and that all of the fine particles clustered together (Figure 5a). Combined with Figure 2 and Figure 3, a large quantity of precipitation occurred because of the high urease activity of the strain, which rapidly hydrolyzed the urea to produce much CO_3_^2−^; thus, Cd^2+^ can be combined with CO_3_^2−^ to form a large number of small mineral crystal diameter precipitates in a short time, resulting in excessive Cd^2+^ binding sites by the bacterial cell. The precipitation mineralized by strain CZW-5 was a spherical shape with a diameter of approximately 2 μm, and a small rod-shaped precipitate was attached to the precipitated large particles (Figure 5b). Bacteria play an important role in the biomineralization of CdCO_3_ because they provide a large number of nucleation sites and hydrolyze the substrate to control the specific morphology of crystals [46]. The precipitation mineralized by strain CZW-9 was also spherical; the largest diameter is approximately 1 μm, and most are smaller than 1 μm (Figure 5c). However, this strain produced significantly fewer crystal nucleation sites than strain CZW-2. In comparison, in the mineralized precipitation of CZW-5, the small-diameter crystals are mostly clustered together to form a large number of small spherical bodies. The mineral precipitate mineralized by strain CZW-12 was similar to those of strains CZW-2 and CZW-9. The crystal volume of the precipitate is small, most of the crystal diameters are less than 1 μm and the precipitates are clustered and piled up (Figure 5d). 

The SEM images of the mineralized product induced by different strains revealed that the diameter of CdCO_3_ ranked from 0.5 to 2 μm. Interactions between bacteria and minerals indicate that minerals provide a place for bacterial activity, and bacteria can promote or inhibit the growth of minerals, resulting in significant differences in morphology, diameter and bulk density of the precipitates [47]. The differences in bacterial strains and urease activity can also lead to differences in the shapes of precipitates [48,49]. Paassen [12] found that the types of calcium carbonate crystals produced by MICP (Microbially Induced Calcite Precipitation) are mainly related to the hydrolysis rate of urea. In addition, the increase of the number of bacteria would increase the precipitation rate of calcium carbonate, because the bacterial cell was the nucleation site of the new crystal precipitation. When more bacteria were present, due to the abundant nucleation sites, CO_3_^2−^ was consumed by the formation of new crystal precipitation, rather than promoting the growth of the existing crystal, which happened in the case of a small number of bacterial cells [50]. This phenomenon has been well studied in the production of calcium carbonate by the pure chemical method, where a large number of nucleation sites leads to greater formation of small crystals, and vice versa [51].

**Figure 6:**
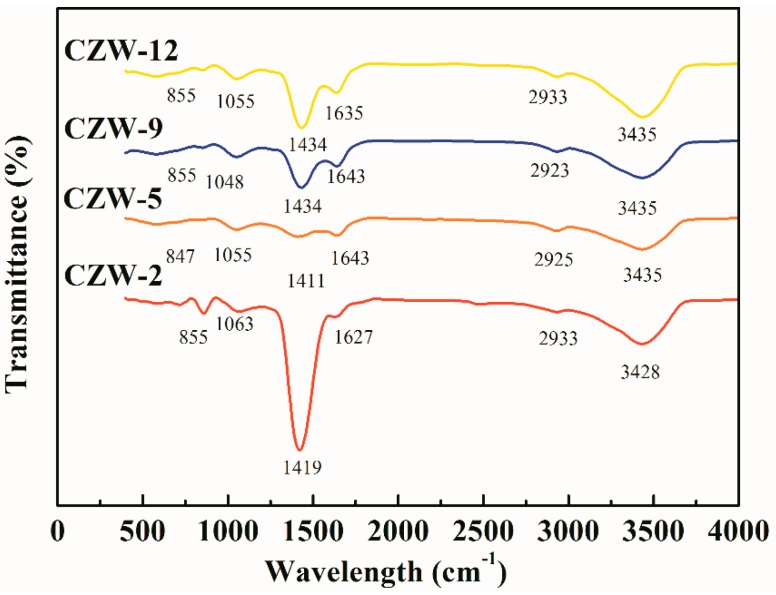
Fourier transform infrared spectroscopy (FTIR) analysis of the precipitates produced by CZW-2, CZW-5, CZW-9 and CZW-12.

To identify the elements in the minerals, the minerals were analyzed by EDS (Figure 5, Spectrum A, B, C, D). The mass percentages of Cd in the precipitations mineralized by strains CZW-2, CZW-5, CZW-9 and CZW-12 were 86.90%, 77.26%, 78.54% and 83.32%, respectively. The mineral precipitates containing heavy metal of CZW-2 and CZW-12 had higher ratios of Cd. Combined with Figure 4, Figure 5 and Figure 6, the absorption frequency of CdCO_3_ and CO_3_^2−^ were CZW-2 > CZW-12 > CZW-9 > CZW-5. These results imply that the mineral crystallinity exhibits a positive correlation with the absorption frequency and is also consistent with the elemental composition of the EDS energy spectrum, but has a negative correlation with the crystal diameter. 

### 3.6. FTIR Spectroscopy

Through Fourier transform infrared spectroscopy analysis, the main functional groups can be determined by obtaining the characteristic peak of microbial mineralization. The peak value of the infrared spectrum of CdCO_3_ precipitation is mainly determined by the internal vibration of CO_3_^2−^, the absorption frequency of CO_3_^2−^ in the infrared region, the strong absorption peak of 1530–1320 cm^−1^, and the weak absorption peaks of 1100–1040 cm^−1^, 890–800 cm^−1^ and 745–670 cm^−1^. The absorption peak located at 3400 cm^−1^ is the stretching vibration peak of O-H and N-H bonds, and the absorption peak of crystal water around 1643 cm^−1^. As shown in Figure 6, the strong absorption peaks in the 1530–1320 cm^−1^ region and the weak peaks in the ranges of 1100–1040 cm^−1^ and 890–800 cm^−1^ are all the C-O bonding of CdCO_3_ crystals [52]. The out-of-plane bending vibration peak occurs at 710.01 cm^−1^, the in-plane bending vibration peak occurs at 862 cm^−1^, and the symmetrical and asymmetrical stretching vibrational peaks occur at 1060 cm^−1^ and 1420.41 cm^−1^, respectively [53,54]. The strong absorption peak that appeared at 1410.2 cm^−1^ was the absorption peak of CO_3_^2−^. The infrared absorption peak intensities of the mineral samples of the four strains decreased from CZW-2 > CZW-12 > CZW-9 > CZW-5, and the mineral crystallinity decreased from CZW-2 > CZW-12 > CZW-9 > CZW-5. Also, the mineral crystallinity exhibits a positive correlation with the crystal peak size of the precipitation in Figure 4 and a negative correlation with the mineral grain size in Figure 5. Compared with the infrared spectra of the CZW-5 mineral precipitation, which exhibited peaks at 847 and 1411 cm^−1^, the strong absorption peaks of the other three precipitates are as follows: CZW-2: 855 cm^−1^, 1411 cm^−1^; CZW-9: 855 cm^−1^, 1434 cm^−1^; CZW-12: 855 cm^−1^, 1434 cm^−1^. These strong absorption peaks exhibited blueshift, which indicates that the carbonate group is more stable. The functional groups on the surfaces of microorganisms act as binding sites for many chemicals, especially trace metals, and also adhere to the surfaces of cells and minerals [55]. Microorganisms can secrete one or more metabolites and combine with chemicals in the system to produce minerals [56].

### 3.7. Cd Analysis after Bioremediation

**Figure 7:**
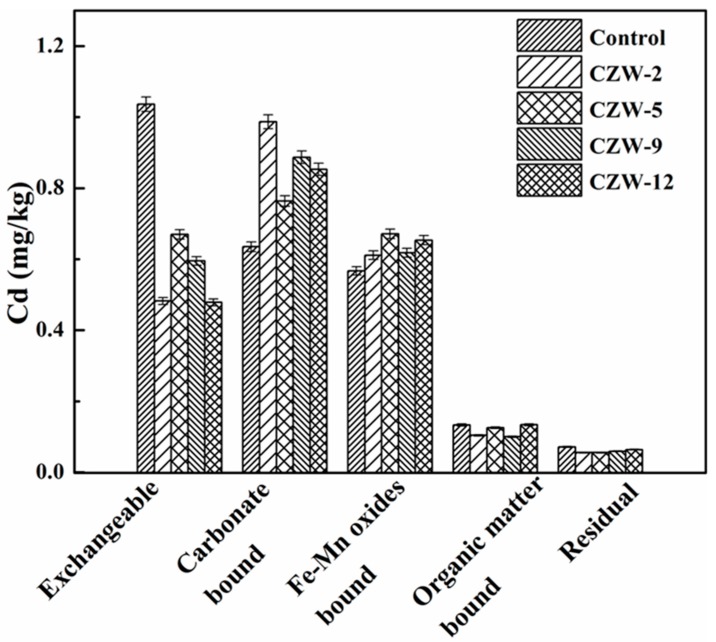
The distribution of Cd in different fractions of the control and bioremediated Cd- contaminated soil.

The bioremediation efficiency of isolated strains was tested in Cd-contaminated soil. Soil samples had an initial total Cd concentration of 5.10 mg/kg. The results of the bioremediation of strains and control after one month are shown in Figure 7. The five fractions in the control soil samples showed the following distribution: exchangeable > carbonate-bound > Fe-Mn oxides-bound > organic matter-bound > residual forms. In the remediated soil samples, the following distribution was obtained: carbonate-bound > Fe-Mn oxides-bound > exchangeable > organic matter-bound > residual forms. Compared to the control samples, the carbonate-bound and exchangeable fractions in the bioremediation soils changed significantly; the carbonate-bound fractions in the bioremediation soils by the mineralization of the strains CZW-2, CZW-5, CZW-9 and CZW-12 increased by 55.42%, 20.27%, 39.67% and 34.36%, while the exchangeable fraction decreased by 53.30%, 35.33%, 42.54% and 53.80%, respectively. However, there was no obvious change in Fe-Mn oxide fraction of Cd in the bioremediation soils and the control. The limited interaction between the isolated four strains and Fe-Mn oxide fractions of Cd may be due to the limited bioavailability [22]. The organic matter bound part of Cd may be complexed or adsorbed as was reported by previous studies [6]. Therefore, the interactions between bacteria and metals are inhibited. The residual fraction of Cd did not change significantly in the bioremediation soils and the control, which, due to the residual fraction of Cd, are tightly bound and very stable under natural conditions [57].

It is thus clear that all four strains can reduce the bioavailability of Cd through immobilizing the Cd from Cd-contaminated soil, but with different degrees of variation in the exchangeable fraction and the carbonate-bound fraction. CZW-2 was more effective in bioremediation soils compared with other three groups, due to the increase in the bacteria cell growth, and the urease activity showed a difference, which coincided with the research by Zhu et al. and Li et al. [8,11]. Zeng et al. [58] found *Cupriavidus* sp. ZSK had high tolerance to heavy metals, such as Cd, Cu, Zn, Cr(VI) and Pb ions, and suggested that *Cupriavidus* sp. ZSK was a potential microorganism to adsorb Cd and other metals from waste water. The remediation by bacterial treatment can alleviate the exchangeable fractions of heavy metals. Thus, the mobility and bioavailability of highly toxic heavy metals can be effectively controlled. Further, the data obtained in this study have provided an effective theoretical basis for soil remediation in large areas.

## 4. Conclusions

In summary, four urease-producing strains (CZW-2, CZW-5, CZW-9, CZW-12) isolated in the abandoned mine were all capable of tolerating high concentrations of Cd and effectively converted soluble Cd into insoluble carbonate minerals. Further, the mineral precipitates from different strains had varied mineral morphologies, diameters and crystallinities, which were significantly correlated with bacterial urease activity. Biomineralization of Cd-contaminated soils with bacteria significantly revealed that the four strains can effectively reduce the exchangeable fraction of Cd but increased the carbonate-bound fraction. Therefore, these four stains can alleviate the mobility and bioavailability of Cd in Cd-contaminated soils. However, this study only describes the results of bioremediation of Cd-contaminated soil obtained during a period of 30 days. In future research, we will focus on the changes in soil properties and morphology after remediation by bacterial treatment. Introduction of this indigenous bacterium provides a potential in situ bioremediation technology without disturbing the target environment, which also provides a new theoretical reference for removing heavy metals in wastewater and soils.
